# Impact of the electron donor on in situ microbial nitrate reduction in Opalinus Clay: results from the Mont Terri rock laboratory (Switzerland)

**DOI:** 10.1007/s00015-016-0256-x

**Published:** 2017-02-24

**Authors:** Nele Bleyen, Steven Smets, Joe Small, Hugo Moors, Natalie Leys, Achim Albrecht, Pierre De Cannière, Bernhard Schwyn, Charles Wittebroodt, Elie Valcke

**Affiliations:** 1 Belgian Nuclear Research Centre SCK•CEN, Boeretang 200, 2400 Mol, Belgium; 2 National Nuclear Laboratory NLL, Chadwick House, Birchwood Park, WA3 6AS Warrington, UK; 3 Agence Nationale pour la Gestion des Déchets Radioactifs Andra, 1-7, Rue Jean-Monnet, 92298 Châtenay-Malabry Cedex, France; 4 Federal Agency for Nuclear Control FANC, Rue Ravenstein 36, 1000 Brussels, Belgium; 5 National Cooperative for the Disposal of Radioactive Waste NAGRA, Hardstrasse 73, 5430 Wettingen, Switzerland; 6 Institut de Radioprotection et de Sûreté Nucléaire IRSN, 31, Avenue de la Division Leclerc, 92260 Fontenay-Aux-Roses, France

**Keywords:** Nitrite, Redox, Clay, Acetate, Hydrogen, Microorganisms, Nuclear waste disposal

## Abstract

At the Mont Terri rock laboratory (Switzerland), an in situ experiment is being carried out to examine the fate of nitrate leaching from nitrate-containing bituminized radioactive waste, in a clay host rock for geological disposal. Such a release of nitrate may cause a geochemical perturbation of the clay, possibly affecting some of the favorable characteristics of the host rock. In this in situ experiment, combined transport and reactivity of nitrate is studied inside anoxic and water-saturated chambers in a borehole in the Opalinus Clay. Continuous circulation of the solution from the borehole to the surface equipment allows a regular sampling and online monitoring of its chemical composition. In this paper, in situ microbial nitrate reduction in the Opalinus Clay is discussed, in the presence or absence of additional electron donors relevant for the disposal concept and likely to be released from nitrate-containing bituminized radioactive waste: acetate (simulating bitumen degradation products) and H_2_ (originating from radiolysis and corrosion in the repository). The results of these tests indicate that—in case microorganisms would be active in the repository or the surrounding clay—microbial nitrate reduction can occur using electron donors naturally present in the clay (e.g. pyrite, dissolved organic matter). Nevertheless, non-reactive transport of nitrate in the clay is expected to be the main process. In contrast, when easily oxidizable electron donors would be available (e.g. acetate and H_2_), the microbial activity will be strongly stimulated. Both in the presence of H_2_ and acetate, nitrite and nitrogenous gases are predominantly produced, although some ammonium can also be formed when H_2_ is present. The reduction of nitrate in the clay could have an impact on the redox conditions in the pore-water and might also lead to a gas-related perturbation of the host rock, depending on the electron donor used during denitrification.

## Introduction

In several countries such as Belgium and France, clay formations are foreseen as host rocks for geological disposal of bituminized intermediate-level long-lived waste (ILW). Suitable clay formations exhibit several favorable hydromechanical and geochemical characteristics (e.g. low permeability, reducing chemical conditions), which delay and spread in time the migration of released radionuclides (De Craen et al. 2004; Andra 2005; Smith et al. 2009). Besides radionuclides, the bituminized radioactive waste under investigation here also contains a high amount of NaNO_3_, dispersed inside a hydrophobic bitumen matrix used to immobilize the waste and serving as a highly efficient semi-permeable membrane. After saturation of the disposal gallery, the bituminized waste will slowly start to take up water, resulting in the dissolution and leaching of NaNO_3_ (Valcke et al. 2009). In addition, soluble organic compounds, initially present or resulting from bitumen degradation, are expected to be released into the clay pore-water (Valcke et al. 2000a, b). Furthermore, the production of H_2_—by anaerobic metal corrosion and by radiolysis of water and bitumen—will be unavoidable in a repository for this type of waste.

**Fig. 1:**
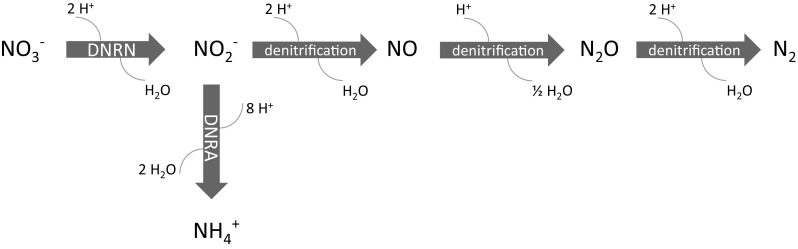
Biological pathways that reduce nitrate include dissimilative nitrate reduction to nitrite (DNRN), to gaseous N species like NO, N_2_O and N_2_ (denitrification) and to ammonium (DNRA). Each of these pathways requires specific reductase enzymes

The release of nitrate could initiate several biogeochemical processes in the clay surrounding the waste disposal gallery, possibly affecting some of the favorable characteristics of the host rock. For example, the nitrate plume could affect the redox conditions (initially reducing) of the host rock in the vicinity of the repository due to microbial nitrate reduction using clay components (e.g. organic matter, pyrite or other Fe(II)-containing minerals) as electron donor (Hauck et al. 2001; Jørgensen et al. 2009; Mariën et al. 2011; Zhang et al. 2012, 2013). As the reducing capacity of the undisturbed clay formation will strongly impact the speciation, the solubility, the retention and the transport properties of redox-sensitive radionuclides (Se, Tc, U, Np, Pu, etc.), clay oxidation might favor the migration of these radionuclides in the host rock (e.g. De Cannière et al. 2010). Furthermore, microbial reduction of nitrate would either lead to the generation of nitrite [by dissimilative nitrate reduction to nitrite (DNRN)], nitrogenous gases (by denitrification) or ammonium [by dissimilative nitrate reduction to ammonium (DNRA)] (Fig. 1) (Madigan et al. 2000). The DNRN pathway usually occurs as an intermediate step in the denitrification process, but can also occur without subsequent denitrification, resulting in the accumulation of nitrite (Almeida et al. 1995; Oh and Silverstein 1999). In turn, the produced nitrite can oxidize the clay both biotically and abiotically (Bleyen et al. 2015, 2016), again possibly resulting in a decrease of the reducing capacity of the clay formation. Continuous denitrification could lead to the formation of a separate gas phase when the concentration of produced N gases would exceed the solubility limit of the gases. This might cause fissuring of the host rock and might thus result in the formation of preferential pathways for radionuclide migration (Mallants et al. 2007; Harrington et al. 2012).

To study the possible geochemical and/or gas-related perturbations induced in the near field of a geological repository for the disposal of nitrate-containing bituminized waste, an in situ experiment, named Bitumen–Nitrate–Clay interaction (BN) experiment, is being performed in the Opalinus Clay in the Mont Terri rock laboratory. This in situ experiment consists of a vertical borehole rigged with downhole equipment containing three packed-off chambers (or intervals) in contact with the surrounding clay and initially filled with artificial pore-water (APW) of the Opalinus Clay. Each interval can be injected, circulated and monitored separately. In this paper, the results of two such injection tests are discussed. For this, two intervals were each injected with APW containing NaNO_3_ and were later on given a pulse of either acetate or H_2_. These tests provide insights into the microbial nitrate reactivity (rate and metabolism) in the presence or in the absence of easily biodegradable electron donors, which are expected to be present in a repository for nitrate-containing bituminized ILW.

## Materials and methods

### Characteristics of the Opalinus Clay at Mont Terri

**Fig. 2:**
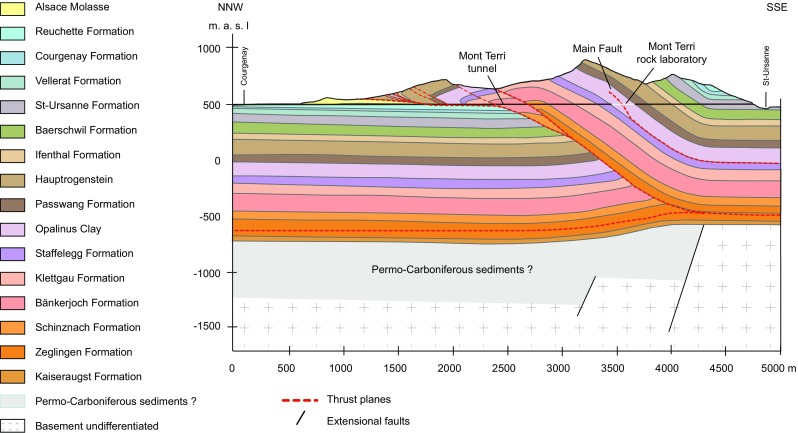
Geological cross-section of the Mont Terri anticline and location of the Mont Terri rock laboratory (Nussbaum et al. 2017)

The Mont Terri Underground rock laboratory is located in the Opalinus Clay, a Mesozoic shale formation (~174 Ma) in the Jura Mountains of North-Western Switzerland. It transects an anticline structure of the folded Jura Mountains and has an overburden of max 320 m (Fig. 2). A detailed overview of the Mont Terri rock laboratory, including the location of the in situ experiment discussed in this paper, is provided by Bossart et al. (2017). The Opalinus Clay at Mont Terri is subdivided into lithological sub-units with shaly, sandy and carbonate-rich sandy facies (Thury and Bossart 1999). The mineral composition of these subunits is rather similar (though not quantitatively) and comprises mainly of quartz, illite and mixed-layer illite–smectite, kaolinite, chlorite, biotite and muscovite, calcite, aragonite, siderite, dolomite and/or ankerite, albite and/or plagioclase, K-feldspar, pyrite, organic matter (mostly kerogen), and other trace minerals like apatite (Pearson et al. 2003).

The water collected in situ from boreholes at the Mont Terri rock laboratory is of the NaCl type with Cl^−^ concentrations ranging from less than 28–310 mM and is characterized by a near-neutral pH (7–8). The chloride concentration in a certain borehole of the Mont Terri can be estimated from its location in the Opalinus Clay (Pearson et al. 2003). Other major components of the Opalinus Clay pore-water are sulfate (concentration fixed based on the Cl^−^ concentration and the ${\text{SO}}_{4}^{2 - }$/Cl^−^ ratio of present seawater), dissolved carbonate species (corresponding to a *p*CO_2_ ranging from 10^−1^ to 10^−2.7^ kPa) and dissolved organic carbon (DOC; usually below 1.7 mmol C L^−1^). As the concentrations of ${\text{SO}}_{4}^{2 - }$ and cations such as Mg^2+^ and Ca^2+^, as well as the alkalinity are all linked to the chloride content and the chloride concentration is depending on the location in the Opalinus Clay (Pearson 1999; Pearson et al. 2003), the chemical composition of the pore-water in the clay surrounding the BN borehole could be estimated based on its location in the clay stratigraphy. From this, the composition of the artificial pore-water used in the BN experiment was derived (Table 1).

**Table 1:** Chemical composition (concentrations in mM) of the artificial pore-water (APW) used to saturate the intervals [target composition derived from Pearson (1999) and Pearson et al. (2003)] and of the interval solutions after saturation of the borehole and equilibration of the surrounding clay for ~8 months

Component	APW	Interval 1	Interval 2	Interval 3
Na^+^	162	174.0	174.0	174.0
K^+^	1.05	1.2	1.2	1.2
Ca^2+^	12.6	11.7	11.0	10.7
Mg^2+^	8.6	9.7	9.5	9.1
Sr^2+^	0.4	0.4	0.4	0.4
Total dissolved Fe	–	n.a.	n.a.	<0.0004
Cl^−^	181	203.1	205.9	203.1
${\text{SO}}_{4}^{2 - }$	12.28	11.03	11.66	11.66
${\text{NO}}_{3}^{ - }$	–	<0.01	<0.01	<0.01
${\text{NO}}_{2}^{ - }$	–	<0.1	<0.1	<0.1
${\text{NH}}_{4}^{ + }$	–	n.a.	n.a.	0.083
CHOO^−^	–	<0.0056	<0.0056	<0.0056
CH_3_COO^−^	–	<0.009	<0.009	<0.009
${\text{C}}_{2} {\text{O}}_{4}^{2 - }$	–	<0.006	<0.006	<0.006
TIC	2.8	5.2	2.8	2.8
TOC	–	0.7	0.92	0.8
pH (−)	7.8	7.1	7.2	7.4

The errors on the concentrations are 4% ([${\text{SO}}_{4}^{2 - }$]), 5.5% ([${\text{NO}}_{3}^{ - }$]), 6% ([Cl^−^]), 10% ([Na^+^], [K^+^], [Ca^2+^], [Mg^2+^], [Sr^2+^], [${\text{NH}}_{4}^{ + }$], [CHOO^−^], [CH_3_COO^-^], [${\text{C}}_{2} {\text{O}}_{4}^{2 - }$] and TIC), 15% ([${\text{NO}}_{2}^{ - }$], [total dissolved Fe]) and 30% (TOC) (95% confidence), while the uncertainty on the pH is estimated to be 0.1 pH unit (95% confidence)

*n.a.* not analyzed

### Design of the BN experiment

#### Borehole general configuration and drilling conditions

**Fig. 3:**
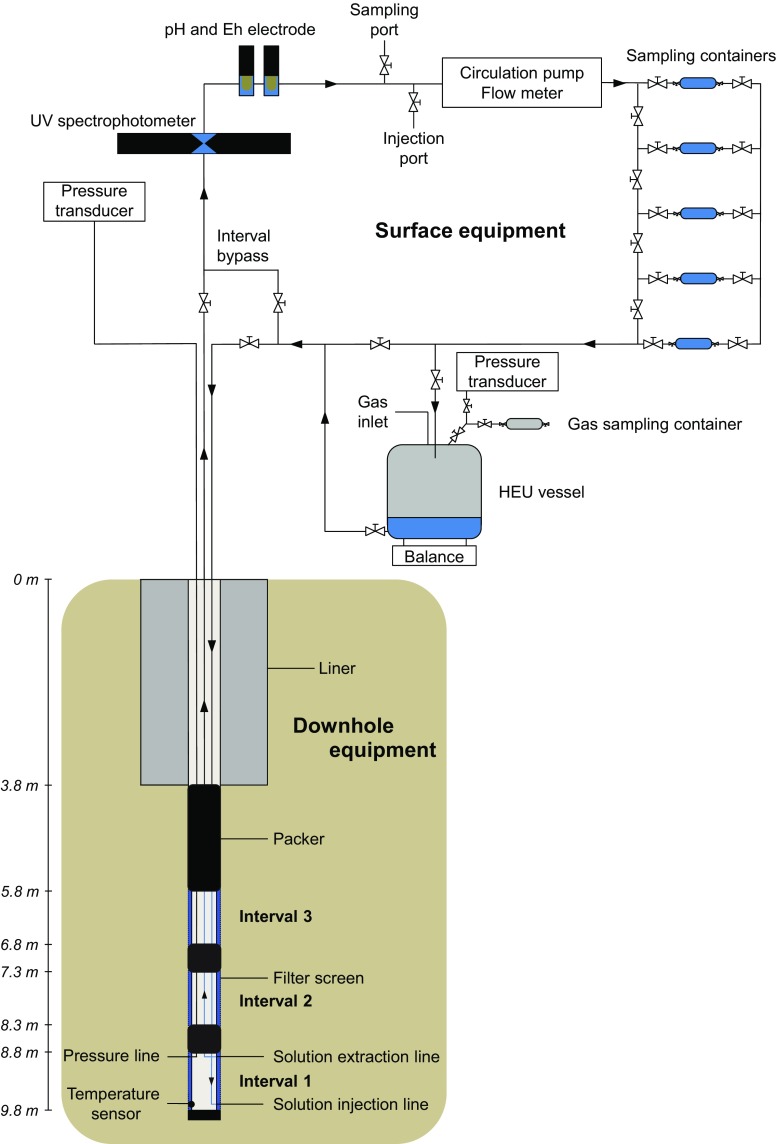
Schematic overview of the BN experiment, consisting of a vertical borehole rigged with a downhole equipment consisting of three packed-off intervals, each lined with a cylindrical sintered stainless steel filter screen to allow contact with the surrounding clay. Each interval is connected to a stainless steel water circulation unit, equipped with a circulation pump, a flow meter and water sampling containers. In two of the intervals, an online UV spectrophotometer and pH and E_h_ electrodes are also installed and in the circulation of Interval 1, also a Hydrogen Equilibration Unit (HEU) is available

In 2010, the BN borehole was drilled vertically in the shaly facies of the Opalinus Clay, in the EZ-A niche of the Mont Terri rock laboratory (location shown by Bossart et al. 2017). It is 9.8 m deep with a diameter of 400 or 76 mm, for the first 3.5 m and the next 6.3 m under the gallery floor respectively (Fig. 3). The small diameter borehole (used for the BN experiment) was drilled under continuous nitrogen flushing, to prevent oxidation of clay components as much as possible and to preserve anaerobic microbial communities possibly present in the clay.

#### BN downhole equipment

The BN downhole equipment consists of three packed-off water chambers (or intervals) of 90 cm long, isolated from each other and from the gallery by inflatable packers. Each interval consists of a cylindrical sintered stainless steel filter screen [porosity 40%; outer diameter (OD) 70 mm, inner diameter (ID) 66 mm] surrounding a central stainless steel casing (OD 60.3 mm). The packer system contains one 2-m long and two 50-cm long hydraulically inflatable packers (Fig. 3) made of a stainless steel central tube surrounded by sleeves with an inner layer of natural rubber and an outer layer of neoprene and reinforced with stainless steel wires. After installation of the downhole equipment, the packers were inflated individually to a pressure of 5 MPa, by injecting water using dedicated stainless steel lines. At this pressure, the packers successfully isolate the intervals from each other and from the gallery. This was confirmed during later injection tests with nitrate: injection of the middle interval (Interval 2) with 1–25 mM of nitrate did not affect the chemical composition of the other intervals (monitored during max 1 year).

Stainless steel water lines (OD 4 mm and ID 2.4 mm) connect each of the downhole intervals to a water sampling unit and an online chemical monitoring unit in the gallery. To optimize water exchange in the intervals, both the injection and the extraction lines are connected as close as possible (i.e. 25 mm) to the opposite interval ends. The injection line is located at the bottom of the interval while the extraction line is installed at the top (Fig. 3). The water pressure in the intervals is measured by automatic pressure transducers (Keller AG für Druckmesstechnik, Switzerland), connected to separate water lines that are also positioned 25 mm from the top of each of the chambers of the downhole equipment. On the bottom of Interval 1 (bottom interval), a temperature sensor (PT1000 type; JUMO, Switzerland) has been placed (Fig. 3).

Geological and structural mapping of the BN drill core indicated the presence of one (pre-existing) fault parallel to the bedding plane close to Interval 1, while no fracturing was found for Interval 2. Five bedding parallel faults are located between 4.4 and 5.5 m below the base of the gallery, which is close to Interval 3 (Phister et al. 2010).

#### BN surface equipment

A schematic overview of the BN surface equipment can be found in Fig. 3.

The water in each of the intervals is continuously circulated from the downhole equipment to the surface equipment and back using a circulation pump (Micropump, USA) combined with a flow meter (ABB, Switzerland). This results in a homogenous solution and allows real-time monitoring of its chemical composition. Furthermore, each test circuit is equipped with 5 sampling containers (40 or 150 mL), which can be removed without causing a perturbation when additional chemical and/or microbial analyses are required (Fig. 3). Additional sampling is also possible by collecting the interval solution into an argon-flushed sterile septum bottle via a needle valve positioned in the water sampling unit (‘sampling port’ in Fig. 3). To prevent fluctuations of the water pressure in the interval as much as possible, the latter sampling method was performed only at the start and at the end of the injection tests, while bypassing the downhole equipment.

To monitor the real-time nitrate and nitrite concentrations, and the redox potential and pH, an UV spectrophotometer and a redox and pH electrode (spectro::lyser™; redo::lyser™ and pH::lyser™ from S::can Messtechnik GmbH, Austria) have been installed in the circulation of two of the test intervals (Interval 1 and 2). When the interval solution is circulating, it flows continuously through the slit of the spectrophotometer, thereby passing between the UV light source and the detector, and through the housing chambers in which the electrodes are immersed. Further information regarding the spectrophotometer and electrodes is given in Sect. 2.6.3.

To investigate the impact of H_2_ on the nitrate reactivity, a Hydrogen Equilibration Unit (HEU) was installed in the circuit of Interval 1 (Fig. 3). In this unit, the circulating interval solution drips into a stainless steel vessel of 10 L, filled with ~1 L of solution and ~9 L of gas. The gas pressure in this vessel is monitored online by a pressure transducer (Keller AG für Druckmesstechnik, Switzerland; range 0–1000 kPa) and controls the water pressure in the interval. Dissolution of the gas into the solution is further maximized by slowly but continuously stirring of the solution. The H_2_ supply tank rests on a balance in order to monitor the weight of the interval solution within the vessel to determine the volume of the solution and the gas phase in the HEU and as such to ensure that the tank does not completely fill with gas. During the injection test with H_2_ (Sect. 2.5), the HEU serves both as a supply of H_2_ and as a gas trap, collecting the gases produced by denitrification. To determine the gas composition in the HEU, a gas sampling container is connected to the HEU vessel. During tests in the absence of H_2_ (e.g. tests described in Sect. 2.5.1), the HEU was not placed in the circuit of Interval 1.

Based on the individual volumes of the equipment (setup as used for the currently discussed tests) and the dimensions of the borehole, an estimation of the initial volume of the solution in the circuit of intervals 1 and 2 could be made, i.e. 3.3 L for Interval 1 and 2.8 L for Interval 2, of which 1.4 L is present inside each interval. Note that the total volumes are depending on the number of sampling containers and/or HEU present in the circuit.

#### Prevention of organic and microbial contamination of the borehole during installation of the experiment

Only stainless steel and neoprene were used as materials for the downhole and gallery surface equipment to avoid biodegradation of the equipment and thus the release and contamination of the borehole water with dissolved organic matter, like was observed in a previous in situ experiment in the Mont Terri rock laboratory (De Canniere et al. 2008, 2011). To prevent additional organic and/or microbial contamination of the borehole as much as possible, all equipment (downhole and in the gallery) was cleaned before installation by rinsing, first with acetone (to remove traces of grease) or ethanol (disinfectant) and later thoroughly with deionized water to remove the cleaning solvents. Before installation, all metallic parts were additionally autoclaved, to prevent microbial contamination as much as possible. Furthermore, the housing chambers of the UV spectrophotometer and electrodes were cleaned with ethanol and subsequent rinsing with deionized water before the start of each new test.

### Saturation and equilibration of the borehole

Immediately after installation of the downhole equipment, the three intervals were injected with APW. Its chemical composition was derived from the location of the BN experiment in the clay stratigraphy and the relationship between sulfate, cations and the chloride content (Pearson 1999; Pearson et al. 2003), though without the organic matter (Table 1). This APW was prepared, stored and injected in the intervals anaerobically (Ar atmosphere with [O_2_] <5 × 10^−4^ vol%) to avoid oxidation of the clay surrounding the borehole. It was injected into the intervals at a water pressure of 300–500 kPa (absolute). Note that no special precautions were taken to avoid contamination with exogenous microorganisms during preparation of the APW.

To achieve full saturation and hydraulic equilibrium of the interval with the surrounding clay, APW was reinjected when the water pressure in the interval(s) decreased below atmospheric pressure and until the water pressure in the intervals remained stable. After the initial injection of Interval 1 with APW, the water pressure rapidly decreased and stabilized at ~250 kPa (absolute). In Interval 2, three consecutive injections were needed until the water pressure stabilized to a pressure of ~130 kPa (absolute). Note that circulation of the solution during the injection tests resulted in a slightly higher water pressure in both intervals.

After static water diffusion into and equilibration with the surrounding clay at a stable water pressure for ~7 months, the APW in each interval was homogenized by circulation at a flow rate of ~5 mL min^−1^ for 4 weeks. Next, each interval solution was sampled and its chemical composition was determined.

### Study of the diffusion-controlled evolution of anionic and neutral tracers

To assess the diffusion of nitrate into the clay surrounding the BN borehole, the diffusive behavior of non-reactive anionic and neutral tracers in the clay was assessed. For this, the solution in Interval 1 and 2 was replaced completely with an anaerobically prepared APW solution containing two tracers: 15.6 mM bromide and 1600 ‰ δ^2^H–H_2_O (final concentrations in the interval). At the same time, nitrate and/or acetate were also injected in both intervals during the first series of injection tests with low nitrate concentrations (described in Sect. 2.5.1, test codes INT1_2011 and INT2_2011a in Table 2).

**Table 2:** Overview of the discussed in situ tests performed for the BN experiment

Test code	Interval	${\text{NO}}_{3}^{ - }$	${\text{NO}}_{2}^{ - }$	CH_3_COO^−^	H_2_ (dissolved)	Start date	Time of e^−^ donor pulse	Sterility solution injected	Parallel injection of tracers
INT1_2011	1	1.5	–	–	–	2011-03-16	No pulse injection	Not sterile	Br^−^ and ^2^H–H_2_O
INT1_2014	1	15	–	–	2.3	2014-10-01	54 days	Sterile	–
INT1_2015	1	15	–	–	1.6	2015-09-23	48 days	Sterile	Br^−^
INT2_2011a	2	1.1	0.8	0.8	–	2011-03-16	No pulse injection	Not sterile	Br^−^ and ^2^H–H_2_O
INT2_2011b	2	1.1	–	0.3	–	2011-05-31	No pulse injection	Sterile	–
INT2_2013	2	25	–	4	–	2013-11-05	70 days	Sterile	–

The indicated concentrations (in mM) of nitrate, nitrite, acetate and dissolved H_2_ are the final concentrations after injection and dilution in the interval

The injected solution was initially pressurized at a higher pressure (~400 kPa) compared to the water pressure in the intervals, possibly resulting in some initial advective loss of solution. After injection, the interval solution was equilibrated overnight at a flow rate of ~7 mL min^−1^. The next day, the water pressure was decreased to more or less the in situ pressure while sampling the interval solutions and re-injecting new solution using the needle valves. Since then, the interval solutions were circulated continuously at a flow rate of ~10 mL min^−1^. Throughout this diffusion experiment, the water pressure in the interval remained more or less stable: 256 ± 40 kPa in Interval 1 and 208 ± 30 kPa in Interval 2. Some fluctuations of the water pressure occurred however due to small changes in the flow rate and to a small water pressure increase (by ~50 kPa) in Interval 2 caused by denitrification after injection of nitrate and acetate during the first series of injection tests (Sect. 2.5.1). Samples of the interval solutions were taken regularly to monitor the decrease of the tracer concentration.

In September 2015, the diffusive behavior of bromide was re-examined in Interval 1, during a second injection test with nitrate and H_2_ (duplicate test for test INT1_2014 but not discussed in this paper; test code INT1_2015 in Table 2). In contrast to the first diffusion test, only the solution in the surface equipment was replaced by a new, sterile APW solution containing NaNO_3_ and ~29 mM bromide. Dilution and overnight equilibration with the remaining interval solution (at a flow rate of ~40 mL min^-1^) resulted in a final concentration of 17.02 mM bromide. During injection and equilibration, the interval solution was pressurized at ~550 kPa, possibly resulting in some advective loss. Afterwards, the water pressure in the interval was decreased to ~370 kPa while sampling the solution and re-injecting new solution. Since then, the interval solution was circulated continuously at a flow rate of ~10 mL min^−1^. During the first ~10 days, the water pressure increased slightly to 404 ± 15 kPa and remained more or less stable afterwards. At the start of the pulse injection with H_2_, the average water pressure in the interval was not affected, although at the time of the pulse, some water pressure fluctuations were observed due to temporary changes in the pressure of the HEU. At the end of the pulse injection with H_2_, the water pressure had increased slightly (to 472 ± 28 kPa) upon switching to an Ar atmosphere in the HEU vessel (see Sect. 2.5.3). To monitor the decrease of the tracer concentration, samples of the interval solution were taken regularly by disconnecting sampling containers.

The diffusion data of the tracers have been modeled with the Generalized Repository Model (GRM) (Small et al. 2008). This model of the BN experiment represents the geometry of Interval 1 and 2 including the volumes of the circulating fluid, the filter and an assumed void space adjacent to the borehole with a 1-dimensional finite difference grid. The Opalinus Clay is represented by 20 model cells that increase exponentially in length from 5 mm for the first cell to 730 mm in the last cell. Each successive cell is 1.3 times the length of the preceding cell and the 20 cells cover a total of 3.16 m of clay. This model configuration was compared with a model of shorter length (10 cm) and a radial diffusion model, configured in PHREEQC (Tournassat et al. 2011), which showed that effects of boundary conditions and radial diffusion were not significant over a 100-day time scale. A porosity of 0.17 was assumed for the Opalinus Clay (Pearson et al. 2003; Van Loon et al. 2004a) and pore diffusion coefficients for the clay were fitted to the bromide and δ^2^H–H_2_O data.

### Injection of Interval 1 and 2 with a nitrate-containing solution

#### Previous injection tests with low nitrate concentrations

In the first series of tests (not discussed in detail in this paper), the biogeochemical evolution of the solution in the intervals was investigated after injection with low concentrations of nitrate (and/or nitrite) (up to 1.5 mM ${\text{NO}}_{3}^{ - }$) with (Interval 2) or without (Interval 1) acetate (details in Table 2, tests INT1_2011, INT2_2011a and INT2_2011b). During the first test in both intervals, tracers were co-injected to perform diffusion tests. Details regarding the injection of the intervals can be found in Sect. 2.4. The C/N ratio of the nitrate and acetate-containing APW solution used in Interval 2 was 0.4 or 0.8 in the 2 consecutive tests in this interval (INT2_2011a and INT2_2011b). The injected APW solutions were prepared anaerobically. In the first test in both Interval 1 and 2 (Table 2), no special precautions were taken to avoid external contamination with microorganisms. For all later tests, a sterile nitrate-containing APW solution was used to inject the intervals, to prevent further externally influenced changes in the microbial community.

The results of these tests indicated that microbial reduction of nitrate and nitrite can occur in the intervals of the BN experiment, using acetate and/or clay components as electron donors. Comparing the evolution in nitrate and nitrite concentrations in the absence or in the presence of acetate, clearly indicates faster reaction rates of microbial nitrate reduction when the system is fueled with acetate. The observed nitrate reduction rates in these preliminary tests are summarized in Table 3. In these tests, nitrate was reduced to nitrite, ammonium and/or nitrogenous gases. Furthermore, high concentrations of nitrate-reducing prokaryotes were detected after injection of the intervals with nitrate, indicating that the nitrate (and nitrite) reduction, observed during all tests, was microbially mediated. More details are given by Bleyen et al. (2011).

**Table 3:** Summary of the average nitrate reduction rates (in mM day^−1^) observed in the in situ tests of the BN experiment, either in the absence or in the presence of additional electron donors, i.e. acetate or H_2_, as indicated in Table 2

Test code	${\text{NO}}_{3}^{ - }$ reduction rate in absence of additional electron donors	${\text{NO}}_{3}^{ - }$ reduction rate in presence of additional electron donors
INT1_2011	0.04	–
INT1_2014	0.02	0.2–0.7
INT2_2011a	–	0.8
INT2_2011b	–	1
INT2_2013	0.02	1.2

#### Injection tests with high nitrate concentrations

In the second series of injection tests, which are discussed in this paper, sterile APW with higher nitrate concentrations was injected in Interval 1 and 2 (Table 2). For these tests, injection of the intervals was performed by replacing the solution in the surface equipment with a new APW solution containing NaNO_3_ while bypassing the downhole equipment. The solutions for injection of Interval 1 and 2 contained 31 and 59 mM NaNO_3_ respectively, and were filter sterilized after preparation. This way, the microbial population present in the intervals would not be disturbed. Care was taken to prevent O_2_ and microbial contamination during injection.

After replacement of the solution in the surface equipment, the solution was circulated (at 40 mL min^−1^) through the interval to mix it with the remaining solution in the downhole equipment, resulting in a dilution of the injected solution. About 10 h after injection (i.e. overnight homogenization of the new with the old solution), samples of the interval solution were taken and the flow rate was decreased to 10 mL min^−1^. This flow rate was maintained during the remainder of the tests. The nitrate concentration after dilution and overnight homogenization is considered to be the starting concentration of the test, i.e. 15 and 25 mM ${\text{NO}}_{3}^{ - }$ for Interval 1 and 2 respectively, as indicated in Table 2. The water pressure in Interval 1 and 2 was 308 and 166 kPa respectively, at the start of the injection tests (after overnight equilibration of the injected solution).

#### Pulse injection with H_2_ or acetate

For the tests in Interval 1 and 2 that are subject to further discussion in this paper (see Sect. 2.5.2), a pulse injection with a certain electron donor (H_2_ and acetate) was performed after a few months of circulation with only nitrate. This way, the impact of an additional electron donor on the microbial nitrate reactivity was monitored in one single test. In Interval 1, the addition of H_2_ was performed by changing the head space in the HEU vessel from argon to 100% H_2_. For this, the head space was repeatedly evacuated to a pressure of ~4 kPa and filled with pure H_2_, while keeping the inlet and outlet valves of the HEU closed. The pressure of H_2_ in the vessel at the start of the pulse was 320 kPa. After restarting the circulation through the HEU, dissolved H_2_ circulated the system together with the interval solution. Based on Henry’s law [with k_H_ = 0.00073 M atm^−1^ at 14 °C (Lide and Frederikse 1995)], the initial concentration of dissolved H_2_ can be calculated: i.e. 2.3 mM dissolved H_2_. This information, combined with the free gas volume in the HEU vessel (9 L) and the total volume of the interval solution at the time of the pulse (estimated to be 2.8 L), allows us to calculate the total initial amount of moles of H_2_ present in the circuit after switching to a H_2_ atmosphere in the HEU: 1.2 mol H_2_ of which 7 mmol H_2_ were dissolved in the circulated solution. This dissolved H_2_ fraction was replenished each time the solution passed through the HEU, but decreased when the partial pressure of H_2_ in the gas phase decreased during the experiment. After ~10 mM of nitrate was reduced (24 days later) H_2_ was removed from the circuit by bypassing the HEU. At this time, the gas phase of the HEU vessel was sampled and subsequently switched again to an argon atmosphere (same method as for the H_2_ pulse).

The pulse injection of acetate in Interval 2 was performed after 70 days by reconnecting one of the sampling containers (40 mL) containing freshly prepared APW with nitrate at the concentration present in the interval at the time of reconnection (i.e. 16 mM ${\text{NO}}_{3}^{ - }$), and 310 mM sodium acetate. After dilution of the added acetate in the remaining interval solution by circulating the solution for 4 h at 40 mL min^−1^, the concentration was ~80 times diluted to a final concentration of 4 mM acetate. The C/N ratio in the interval solution was 0.4. Afterwards, the flow rate was decreased again to 10 mL min^−1^.

Throughout the tests, samples of the interval solution were taken regularly by disconnecting sampling containers. All collected samples were stored at ~4 °C and under anaerobic conditions prior to the chemical and microbiological analyses, to slow down as much as possible any (microbially mediated) reaction. Furthermore, the nitrate and nitrite concentrations and the pH and E_h_ were monitored online, enabling sampling of the interval solution at appropriate times.

### Monitoring of the in situ chemical composition

#### Chemical analyses of sampled solution

The chemical analyses of the sampled solutions were performed as soon as possible after sampling. Subsamples of the solution were taken under an anaerobic atmosphere and analyzed at SCK•CEN by ion chromatography (IC) for ${\text{SO}}_{4}^{2 - }$, Cl^−^, Br^−^, acetate, ${\text{NO}}_{3}^{ - }$ and ${\text{NO}}_{2}^{ - }$, by inductively coupled plasma atomic emission spectroscopy (ICP-AES) for Na^+^, K^+^, Ca^2+^, Mg^2+^, Sr^2+^ and dissolved Fe(total), by ion selective electrode (ISE) for ${\text{NH}}_{4}^{ + }$ and by TOC/TIC analyzer with UV persulfate digestion for TOC (total organic carbon) and TIC (total inorganic carbon). To monitor the diffusion of deuterated water into the surrounding clay, isotope ratio mass spectrometry (relative to the VSMOW standard) was performed by Hydroisotop GmbH (Germany).

#### Analyses of dissolved gases

To detect the presence of certain dissolved gases (N_2_, N_2_O, CO_2_, H_2_, O_2_) in the interval solutions during the tests, the solution was equilibrated with a gas phase, either (1) in a sampling container after disconnection from the circuit (for Interval 2) or (2) in the gas phase of the HEU (for Interval 1).

To equilibrate the dissolved and gaseous gas fractions in a sampling container, the headspace method [adapted from (Xiong et al. 2006)] was applied, followed by micro gas chromatography with a thermal conductivity detector at SCK•CEN for analysis of N_2_O and N_2_ concentrations. For the headspace method, a volume of ~ 10 mL of the solution in the sampling container was removed, while allowing Ar gas to flow into the created headspace. The dissolved gases were equilibrated with the headspace atmosphere during one week at 4 °C (to prevent additional microbial activity) followed by 4 h at 19 °C while sonicating. Afterwards, the gas phase was sampled for analysis. The concentration of dissolved gases in the interval solution was derived using Henry’s law [with k_H_ (N_2_) = 0.00056 M atm^−1^ and k_H_ (N_2_O) = 0.021 M atm^−1^ at 19 °C (Wilhelm et al. 1977)].

To determine the gas composition in the gas phase of the HEU vessel, the head space of the vessel was sampled before replacing the gas in the vessel by argon (at the end of the pulse with H_2_). The gas phase was analyzed by gas chromatography with a thermal conductivity detector (for H_2_, O_2_, N_2_, CO_2_, N_2_O) at Hydroisotop GmbH (Germany). The concentration of the dissolved gases was calculated using Henry’s law applying the following values for k_H_ (at 14 °C): H_2_: 0.00073 M atm^−1^; O_2_: 0.0010 M atm^−1^; N_2_: 0.00052 M atm^−1^; CO_2_: 0.025 M atm^−1^; N_2_O: 0.018 M atm^−1^ (Wilhelm et al. 1977; Lide and Frederikse 1995).

#### Online monitoring of nitrate and nitrite concentrations and pH

A multi-parameter UV spectrophotometer (spectro::lyser™, S::can Messtechnik GmbH, Austria) was used for the high-resolution online measurements of nitrate and nitrite. The spectro::lyser™ records the UV light absorbance in the wavelength range between 200 and 400 nm, and displays the results in real time. The measurement of the nitrate and nitrite concentrations is based on the turbidity-compensated absorbance of 5–7 wavelengths per parameter (between 210 and 245 nm) using an algorithm specifically adapted for BN waters, provided by the manufacturer. Further calibration was performed before each injection test to obtain accurate measurements and long-term stability of the results. In addition, a cross-checked calculation of the nitrite concentration is possible, based on the normalized absorbance at 245 nm. More details are given by Bleyen et al. (in preparation).

To monitor changes in pH and redox potential in the interval solutions, an online pH and E_h_ probe (respectively pH::lyser™ and redo::lyser™, S::can Messtechnik GmbH, Austria) was used. These probes contain a Refex reference system (Refex Sensors Ltd, Ireland), separating the test solution from the Ag/AgCl reference electrode compartment by means of ceramic frit junctions covered with a chemically stable Refex polymeric interface (consisting of a polyvinyl acetate resin and doped with KCl). The stability and long-term performance of this type of electrodes under in situ conditions are discussed in detail by Bleyen et al. (in preparation).

### Evaluation of the microbial community

Microbiological analyses were performed on the sampled solutions to monitor the evolution of the microbial community upon changes in the water composition and to verify the presence of an active microbial community. These analyses included ATP measurements [method according to Wouters et al. (2013)], cultivation-based techniques and DNA-based molecular biology methods.

#### Cultivation-based methods

The metabolic versatility of the bacterial community was determined using a 5-log dilution and threefold biological replicates (i.e. Most Probable Number (MPN) determination) in four different anaerobic cultivation media: (1) LB medium (Bertani 1951) for detection of heterotrophic anaerobic microorganisms; (2) R2A medium (Reasoner and Geldreich 1985) for slow-growing microbes; (3) Medium 63 (Deutsche Sammlung von Mikroorganismen und Zellkulturen) for the cultivation of sulfate-reducing prokaryotes (SRP); (4) N43 medium (Heylen et al. 2006) to detect nitrate-reducing prokaryotes. All media contained activated resazurin (1 mg L^−1^) as redox indicator to verify anoxicity. The inoculated dilutions were incubated at 30 °C for minimally 4 weeks, after which the turbidity and gas formation and/or precipitation of end products in the media was assessed and the microbial concentration was calculated using the method provided by Garthright and Blodgett (2003).

#### DNA-based microbial community analysis

DNA was extracted from the sampled interval solution applying an in-house developed nucleic acid extraction procedure using potassium ethyl xanthogenate as described by Wouters et al. (2013). This DNA was amplified by polymerase chain reaction (PCR) using universal primers for the bacterial 16S rDNA gene. To be able to detect changes in the microbial community during the different phases of the tests, highly conserved primer sequences were chosen, yielding amplicons covering several variables regions of the 16S rDNA gene. Two types of DNA-based microbial community analyses have been applied for the tests described in this paper: Illumina MiSeq high throughput sequencing (Interval 1) and barcoded 454 FLX + high throughput sequencing (Interval 2) (Moors et al. 2015b).

For the solutions sampled from Interval 1, 464-bp fragments covering the variable regions V3 and V4 of the 16S rDNA gene were first amplified by PCR. For this, the 341F and 785R primers described by Klindworth et al. (2012) and extended with the necessary overhanging adapters were used: 341F 5′-TCGTCGGCAGCGTCAGATGTGTATAAGAGACAGCCTACGGGNGGCWGCAG-3′ and 785R 5′-GTCTCGTGGGCTCGGAGATGTGTATAAGAGACAGGACTACHVGGGTATCTAATCC-3′. Details regarding the PCR conditions are described by Klindworth et al. (2012). Afterwards, a DNA sequencing library was prepared by performing a second PCR, linking an index to either side of the amplicons, according to the instructions of LGC (United Kingdom) who subsequently performed the MiSeq Illumina DNA sequencing.

For the solutions of Interval 2, DNA was amplified yielding 1151-bp fragments of the 16S rDNA gene, including the variable regions V3–V6. The primers for this PCR were FLX-1492R (5′-ATGGAACAATGCTGAA-3′) and a series of forward primers consisting of a DNA-annealing part (5′-CTACGGRAGGCAGCAG-3′) linked to a set of barcodes (MID 049-059; according to the instructions of IMGM, Germany). The PCR reaction was carried out in 50 µl volumes containing 50 vol% Phusion 2× master mix (Thermo Fisher Scientific, USA), 0.5 µM of each primer and 50–100 ng DNA template. The following PCR conditions were used: initial denaturation at 98 °C for 2 min, followed by 30 cycles of denaturation (98 °C for 30 s), annealing (52 °C for 30 s) and elongation (72 °C for 5 min), and a final extension step at 72 °C for 5 min. After equimolar pooling of the amplicon mixtures (each with different barcodes), IMGM (Germany) performed the 454 FLX+ high throughput DNA sequencing.

The obtained raw DNA sequences were treated with an in-house developed bioinformatics pipeline, optimized to preprocess the output of different high throughput sequencing methods. This preprocessing included trimming, denoising (NoDe (Mysara et al. 2015a) or IPED (Mysara et al. 2016)) and chimera removal (CATCh (2015b)). Using this software, sequences were clustered into operational taxonomic units or OTU (Schloss and Westcott 2011). Each OTU contains a number of sequences indicating the abundancy of the OTU in the analyzed sample. Each of the consensus OTU DNA sequences was used to find the closest matching bacterial species by BLAST against the GreenGenes database with a minimum of 90% sequence similarity (DeSantis et al. 2006).

## Results

### Chemical composition of solution in the BN borehole

After saturation of the intervals with APW and equilibration with the surrounding clay for ~8 months, solution was sampled from the intervals. Chemical analyses (Table 1) show that the target composition of the APW and thus the theoretical composition of the pore-water in the Opalinus Clay at the location of the BN experiment do not differ significantly from that of the equilibrated interval solutions, except for the TIC measured in Interval 1. The reason for this difference in TIC is yet unknown, but might be due to an elevated presence of carbonates close to Interval 1. Nevertheless, the chemical composition of the water collected from the three intervals is remarkably similar. The composition of the solutions in Intervals 1 and 2 was determined again before starting the injection tests discussed in this paper. This showed no statistical differences with the composition of the first sampled solution apart from a slight increase in the dissolved iron (up to 0.05 and 0.003 mM for Interval 1 and 2 respectively) and a decrease in the sulfate concentration (by ~1 mM for both intervals). These small changes are most likely due to some microbial activity ongoing in the interval before the start of the currently discussed injection tests. Indeed, microbiological analyses confirmed the presence of a large population of sulfate-reducing bacteria (strains from genera *Pseudomonas*, *Gracillibacter*, and *Desulfosporosinus*) in the sampled solution from both intervals before injection with nitrate (Moors et al. 2015b). Furthermore, some ammonium was found in both interval solutions (0.4 mM in Interval 1 and 0.3 mM in Interval 2), which can be explained by biomass degradation (Madigan et al. 2000).

The invariable (main) chemical composition of the interval solutions confirms that geochemical stability was reached in the BN borehole after 8 months of equilibration with the surrounding clay. Furthermore, the composition of the pore-water in the Opalinus Clay can indeed be deduced with an acceptable accuracy based on its location and the relationship between sulfate, cations and the chloride content (Pearson 1999; Pearson et al. 2003).

The results also revealed that some organic matter had diffused into the intervals during saturation, resulting in a TOC concentration of ~0.8 mmol C L^−1^ for each interval, which is similar to the TOC concentrations measured previously in boreholes in the Opalinus Clay at Mont Terri, i.e. 0.3–3.7 mmol C L^−1^ (Pearson 1999; Courdouan et al. 2007; Eichinger et al. 2011). According to previous studies made on clay aqueous extracts (Courdouan et al. 2007; Eichinger et al. 2011), the low molecular weight (and thus easily biodegradable) organic fraction found in Opalinus Clay water contains highly variable concentrations of acetate (2–31% of DOC), formate (0.2–4% of DOC) and other small organic compounds. In contrast to these studies, no detectable concentrations of acetate, formate or oxalate could be found in the BN interval solutions after the saturation and equilibration period (Table 1) nor before the start of the injection tests discussed here.

### Diffusion-controlled evolution of tracers

The diffusive transport of an anion and a neutral species into the surrounding clay was studied in 2011 by adding bromide and deuterated water to APW injected in Interval 1 and 2 and monitoring the decrease in concentration in the interval solution as a function of time over a period of up to 433 days. Later on, a second bromide injection test was performed in Interval 1 (in 2015), in which the bromide concentration was monitored during 165 days.

**Fig. 4:**
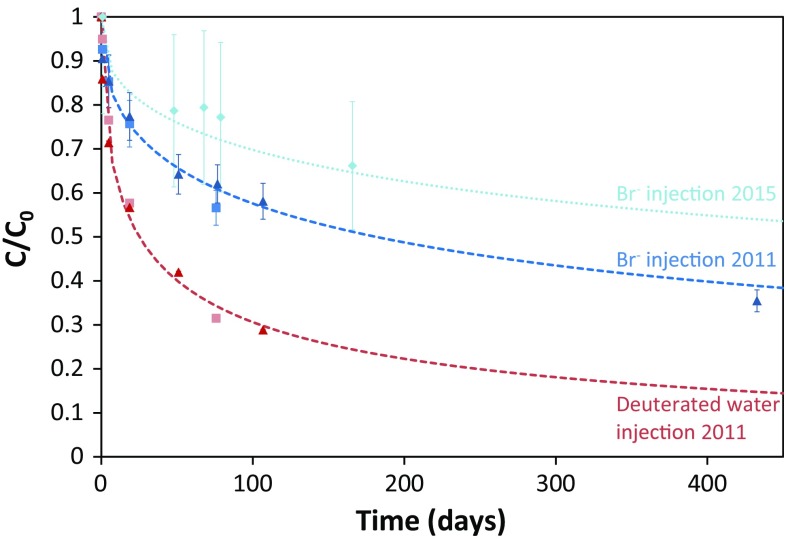
Evolution of the bromide (*blue*) and deuterated water (*red*) concentration (represented in relative concentrations or C/C_0_) measured in the water in Interval 1 (*triangles*) and 2 (*rectangles*) during the first tracer diffusion tests in 2011. For Interval 1 a second bromide test was performed in 2015 (*light blue diamonds*). The expanded error (95% confidence) on the relative concentrations of bromide and deuterated water is, respectively, 6–22% and 0.55%. The *dashed lines* indicate the modeled concentrations based on a pore diffusion coefficient of bromide (2 × 10^−11^ m^2^ s^−1^) and of deuterium (1.2 × 10^−10^ m^2^ s^−1^), obtained based on the data from the first tracer diffusion tests in 2011. The *dotted line* indicates the modeled concentration for bromide based on the pore diffusion coefficient derived from the 2nd bromide diffusion test in 2015 (1 × 10^−11^ m^2^ s^−1^)

The tracers show a steady decrease in relative concentration, which is similar for both intervals. This is consistent with the diffusion of these species in the surrounding clay, as the decrease in relative concentrations in the interval solution is linear with the square root of time after an initial equilibration period (~1 day). Furthermore, the evolution of the tracer concentrations in time can be successfully fitted using the GRM and assuming only diffusion. This indicates that diffusive equilibration of the interval solution and the pore-water in the surrounding clay occurred without a significant contribution of advective transport [more details by Small (2015)]. The results of the first diffusion test also confirm the expected slower diffusion of bromide into the clay compared to deuterated water (Fig. 4), due to anion exclusion, i.e. the derived pore diffusion coefficient of deuterated water was 1.2 × 10^−10^ m^2^ s^−1^, while that of bromide was 6 times lower (2 × 10^−11^ m^2^ s^−1^). Both diffusion coefficients are in the range of coefficients for, respectively, neutral and anionic species determined previously for Opalinus Clay, perpendicular and parallel to the bedding plane (Van Loon et al. 2004a, b; Alt-Epping et al. 2008; Wersin et al. 2008). Nevertheless, compared to pore diffusion coefficients estimated for the Pore-water Chemistry (PC) experiment in the Mont Terri rock laboratory, i.e. 2 × 10^−10^ m^2^ s^−1^ for deuterated water and 9 × 10^−11^ m^2^ s^−1^ for bromide (Alt-Epping et al. 2008; Tournassat et al. 2011) the pore diffusion coefficients for bromide and deuterium in the BN experiment appear to be slightly lower. The reason for this difference is not fully understood and may reflect the location of the BN experiment close to the sandy facies of the Opalinus Clay.

A second injection of Interval 1 with bromide in 2015 indicates that the diffusion rate of bromide has slowed down compared to the initial tests (Fig. 4). The pore diffusion coefficient of bromide derived from this test is 1 × 10^−11^ m^2^ s^−1^. It is possible that this decrease in diffusion coefficient is a result of clogging of the filter screen either by creep of the Opalinus Clay, or as a result of microbial growth or mineral alteration resulting from previous nitrate reactivity in the interval. As the nitrate injection tests discussed in this paper were also performed 2–3 years after the first diffusion test, such clogging could have occurred in the meantime. Moreover, fitting of the nitrate evolution of these injection tests indicated indeed a slower diffusion of nitrate than expected based on the pore diffusion coefficient derived in the first diffusion test with bromide. Therefore, the diffusive behavior of anions in the intervals during the currently discussed tests is assumed to be similar to the behavior of bromide during the second diffusion test. Hence, the pore diffusion coefficient derived for bromide measured in 2015 (1 × 10^−11^ m^2^ s^−1^) has been used to model the reactive transport behavior of nitrate and nitrite anions in the intervals during these injection tests.

### Nitrate reactivity in the BN borehole

#### Microbial community

The microbiological analyses performed on the solutions sampled after saturation and equilibration of the intervals have revealed the presence of an active and versatile microbial population in the BN intervals, before the start of the injection tests. As no special precautions were taken to avoid contamination of the APW with exogenous microorganisms, this population could have been introduced during the first injection or installation of the downhole equipment.

ATP measurements suggest that 2 × 10^5^ to 7 × 10^5^ equivalents of active cells (EAC) per milliliter solution were present in all three intervals after saturation (Moors et al. 2012), based on the estimation that most bacterial cells contain 3 mM of ATP (Neidhart 1996). In addition, MPN analyses show the presence of viable microorganisms in every type of tested MPN medium, but especially prokaryotes capable of reducing nitrate (5 × 10^4^ cells mL^−1^ or more). Autotrophic growth capacity is present but oligotrophic heterotrophy appears to be the preferred carbon metabolism of the residing communities in the BN borehole after saturation with APW.

After injection of Interval 1 and 2 with, respectively, 15 and 25 mM NaNO_3_, a large population of nitrate-reducing bacteria had developed in both intervals. These nitrate-reducing microorganisms included predominantly *Pseudomonas* strains but also strains from the genera *Cupriavidus, Pelomonas, Undibacterium, Acidovorax, Phenylobacterium, Brevundimonas* and *Corynebacterium*. When an easily oxidizable electron donor, such as acetate or H_2_, was supplied as well, the microbial population changed towards a community consisting predominantly of species who prefer using such electron donor, e.g. species from genera *Acidovorax* (after pulse of acetate) and *Clostridium* (after pulse of H_2_) (Moors et al. 2015a). These results indicate that the observed nitrate reduction in the intervals is (mainly) microbially mediated and that changes in the chemical composition of the interval solution are rapidly responded to by a shift in the microbial community.

#### Evolution of the nitrogenous species

**Fig. 5:**
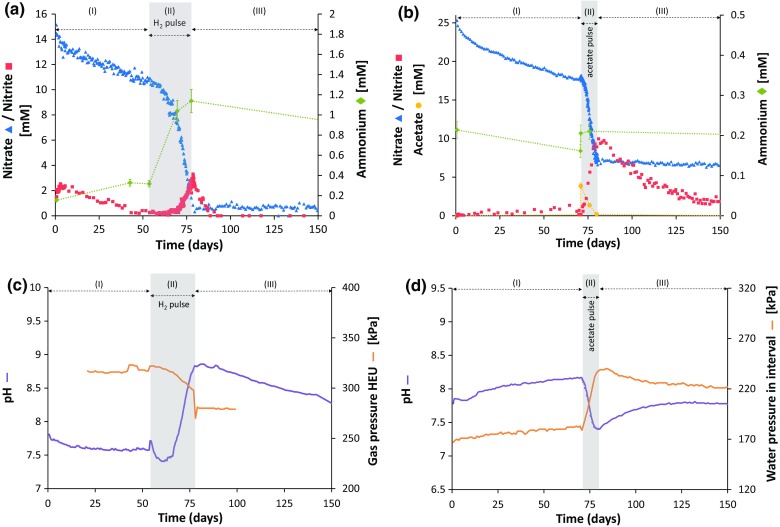
Evolution of the nitrogenous species, pH and pressures after injection of Intervals 1 and 2 with nitrate during tests INT1_2014 and INT2_2013 (Table 2). **a**, **b** Evolution of nitrate (*blue*), nitrite (*red*) and ammonium (*green*) concentrations after injection of **a** Interval 1 with 15 mM ${\text{NO}}_{3}^{ - }$ only and pulse injection with H_2_ after 54 days or **b** Interval 2 with 25 mM ${\text{NO}}_{3}^{ - }$ only and pulse injection with acetate after 70 days. **c** Evolution of the pH (*purple*) and gas pressure in the HEU (*orange*) after injection of Interval 1. **d** Evolution of the pH (*purple*) and water pressure (*green*) in Interval 2. Stages I and III: nitrate reactivity without additional electron donor; Stage II: nitrate reactivity during pulse injection with either H_2_ (Interval 1) or acetate (Interval 2). The errors on the values are 1% (pH), 10% ([${\text{NH}}_{4}^{ + }$], [CH_3_COO^−^]), 7–15% ([${\text{NO}}_{3}^{ - }$]) and 15–20% ([${\text{NO}}_{2}^{ - }$]) for a 95% confidence interval. The uncertainties (95% confidence) on the pressures are 2 kPa (gas pressure) and 30 kPa (water pressure)

**Fig. 6:**
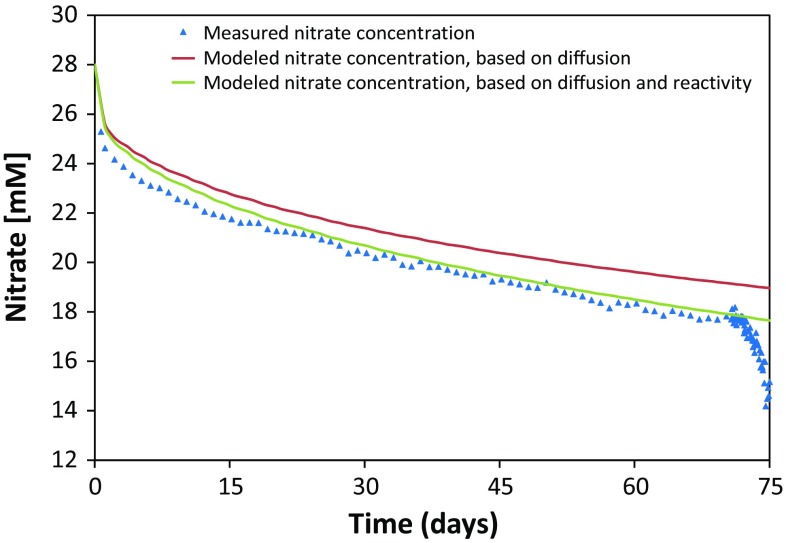
Comparison of measured (*blue triangles*) and modeled concentrations of nitrate after injection in Interval 2 during stage I. The modeled results were obtained using the pore diffusion coefficient of bromide (1.0 × 10^−11^ m^2^ s^−1^), derived from the second bromide tracer diffusion test carried out in 2015. Either only diffusion (*red*) or a combination of diffusion and nitrate reactivity (*green*) were taken into account in the model to fit the measured data

When no additional electron donors were added (stage I in Fig. 5a, b), the nitrate concentration decreased only slowly in time after injection, more or less at the same rate in Interval 1 and 2. This slow decrease in nitrate concentration was mainly controlled by diffusion into the surrounding clay. Indeed, based on the pore diffusion coefficient derived for bromide (1 × 10^−11^ m^2^ s^−1^; see Sect. 3.2), an estimation of the diffusion of nitrate from Interval 2 could be made (Fig. 6). The modeled data show that for this interval only ~11% of the total decrease of the nitrate concentration after equilibration of the interval solution was due to nitrate reactivity. A similar behavior was observed for Interval 1 (data not shown). Based on these modeled results, the reaction rate during stage I was similar in both intervals, i.e. 0.02 mM ${\text{NO}}_{3}^{ - }$ per day (Table 3).

The results of the chemical analyses indeed show that some microbial nitrate reduction has occurred during stage I: nitrite was produced in both intervals, though at a different rate. In Interval 1 (Fig. 5a), ~2 mM ${\text{NO}}_{2}^{ - }$ was produced through DNRN in the first few days after nitrate injection, with most of it being produced during equilibration of the injected solution with the remaining interval solution. On the other hand, in Interval 2 (Fig. 5b) a slower production of nitrite had occurred: during the first 70 days, 0.5 mM ${\text{NO}}_{2}^{ - }$ was produced. After this initial reaction in Interval 1, the nitrite concentration decreased slowly (~0.05 mM per day), until no more nitrite was detected (<0.1 mM) in the interval 54 days after injection (Fig. 5a). Similar to nitrate, this decrease can be attributed to diffusion into the clay, combined with nitrite reduction with natural electron donors. In neither of the intervals was ammonium produced, indicating that DNRA did not occur during this stage (Fig. 5a and b).

To assess the effect of easily oxidizable electron donors on the nitrate reduction, a pulse injection with either acetate (Interval 2) or H_2_ (Interval 1) was performed (stage II in Fig. 5). About 2 days after pulse injection of Interval 2 with 4 mM acetate (Fig. 5b), the nitrate concentration decreased much faster (1.2 mM ${\text{NO}}_{3}^{ - }$ per day) than during stage I, indicating that the microbial nitrate reduction rate was strongly enhanced due to the addition of acetate. During this fast decrease in nitrate concentration, the nitrite concentration increased strongly and both dissolved N_2_O and N_2_ were detected in the interval solution (Fig. 5b; Table 4). The observed increase in the water pressure confirms a fast production of gaseous compounds (Fig. 5d). When acetate was completely consumed in Interval 2 (Fig. 5b; end of stage II), ~ 90% of the nitrate present at the start of the pulse injection was reduced to nitrite (DNRN), while the remainder was likely consumed through denitrification and/or assimilation of N into biomass. Due to the high reaction rate, non-reactive transport of nitrate into the clay is considered to be less important.

**Table 4:** Total amount of gases (in mmoles) present in Interval 2 after complete consumption of acetate (after 78 days) and in Interval 1 after pulse injection with H_2_ (after 78 days)

Gas species	Interval 1		Interval 2
	In solution	In gas phase	
N_2_	0.012	3.5	1.7
N_2_O	<0.2	<0.03	0.37
H_2_	5.5	1.1 × 10^3^	n.a.
O_2_	<0.004	<0.6	n.a.
CO_2_	0.1	0.02	n.a.

For Interval 1, the amount of gases present in solution and in the gas phase of the HEU is given. Due to the absence of a gas phase in Interval 2, the total amount of gases is only represented by the amount of dissolved gases in the interval solution

*n.a.* not analyzed

After a pulse of H_2_ (initially 1.2 mol of H_2_ of which 7 mmol dissolved H_2_ in interval solution) in Interval 1, no changes in the nitrate decrease rate nor in the concentration of reduced N species were observed during the first few days (Fig. 5a; stage II). However, after 5 days the nitrate concentration started to decrease at a higher rate: during the first 9 days of the reaction at ~0.2 mM ${\text{NO}}_{3}^{ - }$ per day and later on at a faster rate (~0.7 mM ${\text{NO}}_{3}^{ - }$ per day). This maximum reaction rate is similar, although slightly lower, to the reaction rate in Interval 2 when acetate was injected. The reaction ceased when H_2_ was removed from the circuit by bypassing the HEU. At the end of stage II, nitrite, ammonium and N_2_ were detected in Interval 1 and in the gas phase of the HEU (Fig. 5a, c; Table 4), indicating that DNRN, nitrate reduction to ammonium, and denitrification had occurred. Production of N_2_O during denitrification was not detected in the head space of the HEU vessel (Table 4). After 78 days, when H_2_ was removed from the circuit, ~34% of the nitrate present at the start of the H_2_ pulse was reduced to nitrite (DNRN) and 8% to ammonium, while the remainder of the fast decrease in the nitrate concentration can be attributed to denitrification and/or assimilation. Again, non-reactive transport of nitrate into the clay can be considered of less importance due to the high nitrate reduction rate after addition of H_2_.

After complete consumption of H_2_ (Interval 1) or acetate (Interval 2), the decrease in the nitrate concentration slowed down considerably (i.e. nearly stopped), as shown in Fig. 5a and b (stage III). At that time, the nitrite concentration had reached its maximal concentration and started to decrease at a rate of 0.2–0.3 mM ${\text{NO}}_{2}^{ - }$ per day in both Interval 1 and 2. This rate is faster than by diffusion only (based on the diffusive behavior of bromide) and therefore must have been caused by nitrite reduction. As nitrite can also be chemically reduced by dissolved organic matter (DOM) or pyrite (Bleyen et al. 2015, 2016), both a microbially mediated and a chemical reaction may have occurred simultaneously.

#### Consumption of electron donors

**Fig. 7:**
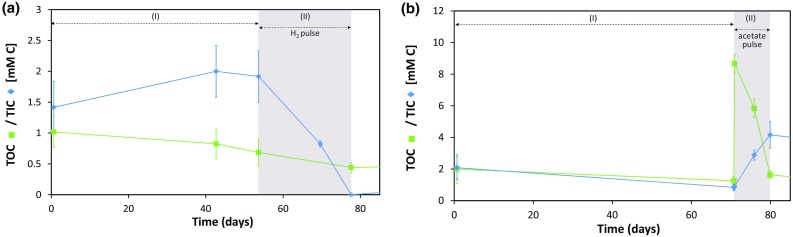
Evolution of the TOC (*green*) and TIC (*blue*) concentrations in Interval 1 and 2 after initial injection with ${\text{NO}}_{3}^{ - }$ only and pulse injection with H_2_ (**a** Interval 1; pulse after 54 days) or acetate (**b** Interval 2; pulse after 70 days). *Open markers* indicate concentrations below detection limit, i.e. 0.4 mmol C L^−1^ TIC. The *error bars* indicate the 95% confidence intervals

In the absence of easily oxidizable electron donors added to the intervals, host rock electron donors such as DOM, pyrite and/or other Fe^2+^ containing minerals have likely been oxidized during the observed reduction of nitrate (stage I) and nitrite (stage III). However, the geochemical data currently available do not allow us to select a hypothesis regarding these electron donors: no significant changes in TOC and TIC (Fig. 7) or in total dissolved iron, thiosulfate or sulfate concentration (data not shown) could be observed during these experimental stages.

After pulse injection of Interval 2 with acetate (Fig. 5b; stage II), the acetate concentration started to decrease rapidly, indicating that this compound was used as electron donor for the observed microbial nitrate reduction. This was expected as acetate is readily available for microbial consumption (Madigan et al. 2000) and its oxidation results in a high energy yield. Based on the production of reduced N species, the following reactions likely have occurred in Interval 2 after pulse injection with acetate [Eqs. (1)–(3); derived from Madigan et al. (2000)]:

1 $${\text{CH}}_{ 3} {\text{COO}}^{ - } {\text{ + 4 NO}}_{ 3}^{ - } \to 4 {\text{NO}}_{ 2}^{ - } {\text{ + 2 HCO}}_{ 3}^{ - } {\text{ + H}}^{ + } \quad \Delta G^{{0^{\prime}}} ({\text{pH}}\; 7) = - 69 \;\frac{\text{kJ}}{{{\text{mol e}}^{-} }},$$

2 $${\text{CH}}_{ 3} {\text{COO}}^{ - } {\text{ + 2 NO}}_{ 3}^{ - } {\text{ + H}}^{ + } \to {\text{N}}_{ 2} {\text{O + 2 HCO}}_{ 3}^{ - } {\text{ + H}}_{ 2} {\text{O }}\quad \Delta G^{{0^{\prime}}} ({\text{pH}}\; 7) = - 86\; \frac{\text{kJ}}{{{\text{mol e}}^{-} }},$$

3 $$5 {\text{CH}}_{ 3} {\text{COO}}^{ - } {\text{ + 8 NO}}_{ 3}^{ - } {\text{ + 3 H}}^{ + } \to 4 {\text{N}}_{ 2} {\text{ + 10 HCO}}_{ 3}^{ - } {\text{ + 4 H}}_{ 2} {\text{O }}\quad \Delta G^{{0^{\prime}}} ({\text{pH }}\;7) = - 101\;\frac{\text{kJ}}{{{\text{mol e}}^{-} }}.$$

The consumption of acetate is also confirmed by the evolution of the TIC and TOC concentrations (Fig. 7):

1. The TOC concentration decreased rapidly in Interval 2 during stage II (by ~7.1 mmol C L^−1^), confirming the consumption of organic C. This decrease is in agreement with the expected TOC decrease based on the acetate consumption (~7.5 mmol C L^−1^; Fig. 7b).
2. A fast increase in the TIC concentration (~3.3 mmol C L^−1^) can be observed in Interval 2 during acetate consumption, which is in agreement with reactions 1–3. However, based on the decrease in acetate, the TIC should have increased by ~7.5 mmol C L^−1^. Most likely, the remainder of the inorganic carbon has been released as gas (CO_2_) and/or precipitated as calcium carbonate. Indeed, the calcium concentration tended to decrease (by ~1 mM; data not shown) during the consumption of acetate and related production of bicarbonate.

Based on the stoichiometry of the reactions and the amount of nitrite and gaseous N species which have been produced, 3.1 mM acetate (or 82% of the total initial acetate concentration in the interval) was used as electron donor. The remainder of acetate could have been used for biomass production. Non-reactive transport of acetate into the clay is considered to be less important due to the fast reaction rate.

In Interval 1, the gas pressure in the HEU (Fig. 5c; stage II) and the partial pressure of H_2_ decreased considerably during the fast nitrate reduction after introduction of H_2_ in the circuit, confirming the consumption of H_2_ as the electron donor. The preferential use of H_2_ compared to naturally present electron donors is also in agreement with the high amount of energy yielded during hydrogenotrophic nitrate reduction. Based on the produced N species, the following microbial reactions are expected to have taken place in Interval 1 after pulse injection with H_2_ [Eqs. (4)–(6); derived from Madigan et al. (2000)]:

4 $$2 {\text{NO}}_{ 3}^{ - } {\text{ + 2 H}}^{ + } {\text{ + 5 H}}_{ 2} \to {\text{N}}_{ 2} {\text{ + 6 H}}_{ 2} {\text{O}}\quad \Delta G^{{0^{\prime}}} ({\text{pH}}\;8) = - 111\; \frac{\text{kJ}}{{{\text{mol e}}^{-} }},$$

5 $${\text{NO}}_{ 3}^{ - } {\text{ + H}}_{ 2} \leftrightarrow {\text{NO}}_{ 2}^{ - } {\text{ + H}}_{ 2} {\text{O}}\quad \Delta G^{{0^{\prime}}} ({\text{pH}}\; 8) = - 81\;\frac{\text{kJ}}{{{\text{mol e}}^{-} }},$$

6 $${\text{NO}}_{ 3}^{ - } {\text{ + 2 H}}^{ + } {\text{ + 4 H}}_{ 2} \to {\text{NH}}_{ 4}^{ + } {\text{ + 3 H}}_{ 2} {\text{O}} \quad \Delta G^{{0^{\prime}}} ({\text{pH}}\; 8) = - 73\;\frac{\text{kJ}}{{{\text{mol e}}^{-} }}.$$

According to the decrease in the gas pressure (Fig. 5c), the composition of the gas phase in the HEU vessel after stage II (Table 4) and the amount of dissolved H_2_ in the Interval 1 solution, the total amount of H_2_ in the interval has decreased by 107 mmol during stage II. Based on the stoichiometry of reactions 4–6, the amount of H_2_ that is used as electron donor to produce ${\text{NO}}_{2}^{ - }$, N_2_ and ${\text{NH}}_{4}^{ + }$, is ~40 mmol. The discrepancy between both values can be explained by either (or both): (1) Some leaks of H_2_ in the equipment or borehole; (2) The large combined uncertainty on the amount of H_2_ that is used as electron donor, which includes the large uncertainties on the calculated volume of the interval solution and of the gas phase. In addition, part of the produced N species have likely been missed, due to diffusion (nitrite, gaseous N species) or sorption (ammonium) onto the clay.

The hydrogenotrophic metabolism in Interval 1 also seems to be confirmed by the decrease in the TIC concentration (decrease ≥1.5 mmol C L^−1^; Fig. 7a), as hydrogenotrophs are generally autotrophs who fix inorganic carbon into biomass (Madigan et al. 2000).

#### Evolution of pH and redox potential

During stage I, the evolution of the pH in both intervals seems to differ slightly (Fig. 5c, d), possibly linked to the observed differences in the microbial reactions prevailing in each interval. In Interval 1, the pH decreased in the first weeks after injection until it reached a stable value at a pH of ~7.6 when the H_2_ pulse was performed (Fig. 5c). On the other hand, in Interval 2, a small but steady increase in pH (total increase of 0.3–0.4 pH units) was observed during the first 70 days of the test (Fig. 5).

When easily oxidizable electron donors were added to the intervals, the pH evolution was highly contrasting between Interval 1 and 2 as the pH evolved in opposite directions, depending on the microbial reactions at work. During the fast microbial nitrate reduction with acetate observed in Interval 2 (stage II), a clear and fast decrease in pH was observed (by ~0.75 pH units; Fig. 5d), which is in agreement with the observed dominant microbial reaction, i.e. DNRN [Eq. (1)]. When the gas in the HEU vessel in Interval 1 was replaced by H_2_, the pH first increased by 0.1 pH units (Fig. 5c), most likely because of the CO_2_ degassing from the solution upon removal of CO_2_ present in the Ar gas phase equilibrated with the interval solution. Afterwards, the pH initially decreased slightly during the lag phase until it reached a new equilibrium. As from the start of the fast hydrogenotrophic reactions, the pH increased significantly (by ~1.4 pH units in total; Fig. 5c), which is corresponding to reactions 4 to 6 and with autotrophic CO_2_ consumption. After complete consumption of the added electron donors (stage III), the initial pH of the interval solutions was slowly regained.

The redox potential of the interval solutions was monitored online as well, providing data on the evolution of the mixed redox potential, i.e. the resultant potential of all redox couples operating in these solutions (Bohn 1971). After pulse injection with acetate, the measured redox potential of the solution in Interval 2 increased by ~100 mV during the fast microbial nitrate reduction to mainly nitrite (from +38 to +140 mV with respect to the standard hydrogen electrode). In contrast, in Interval 1, the addition of H_2_ resulted in a severe decrease of the measured redox potential, i.e. a decrease of ~470 mV (from +97 to −372 mV with respect to the standard hydrogen electrode). As the theoretical redox potential of the H_2_/2H^+^ redox couple is −489 mV (at pH 8 and at a partial pressure of 320 kPa), the decrease in the measured redox potential can almost entirely be attributed to the addition of H_2_ to the system. During the pulse of H_2_, the measured redox potential did not vary significantly (± 20 mV), although also in this test a considerable amount of nitrite was produced. This suggests that the mixed redox potential in the solution of Interval 1 was largely controlled by the H_2_/2H^+^ redox couple due to the rather large quantities of H_2_ in the system. Any changes in the redox potential due to microbial nitrate reduction could therefore not be observed.

## Discussion

### Nitrate reactivity without addition of electron donors

Without addition of electron donors, a slow microbial nitrate reduction was observed, likely using electron donors present in the interval solution or in the surrounding clay (DOM, pyrite, or other Fe^2+^ containing minerals). However, the current chemical data of our experiments do not allow us to determine which electron donor was used during this slow reaction. As a previous study of the microbial community and metabolic activity in borehole water in the Opalinus Clay using metagenomics suggested the use of sedimentary organic carbon as electron donor by sulfate reducers (Bagnoud et al. 2016), further investigation of the microbial community present in the intervals of the BN experiment might provide more insights into the observed slow microbial nitrate reduction.

The slow nitrate reactivity is in agreement with the results of previously reported lab tests during which nitrate reduction was studied in clay slurries or waters (Boom Clay or Callovo-Oxfordian Clay) or with individual clay electron donors (e.g. pyrite, DOM) (Libert et al. 2011; Mariën et al. 2011; Bleyen et al. 2015, 2016). Furthermore, the observed in situ reaction rate is similar to the rate observed during previous injection tests in the BN experiment, e.g. 0.04 mM ${\text{NO}}_{3}^{ - }$ per day after injection of Interval 1 with 1.5 mM NaNO_3_ (code INT1_2011; Tables 2 and 3). In the absence of a sufficient amount of easily oxidizable electron donors, the microbial activity seems thus to be limited by the bioavailability and biodegradability of the electron donors and the rate at which electrons can be provided by the surrounding clay.

In both Interval 1 and 2, nitrate was reduced mainly to nitrite (DNRN), though at a different rate. Furthermore, some nitrite was slowly reduced in Interval 1. The strongly contrasting evolution of the pH in both intervals seems to confirm the previously discussed small differences in metabolic pathways and rates. These differences between Interval 1 and 2 during stage I are likely due to the history of both intervals or to small heterogeneities in the clay formation surrounding each of the intervals, which could have caused small differences in the microbial population present in the interval, and in the availability of electron donors.

### Impact of additional electron donors on the in situ nitrate reactivity

In the presence of acetate and H_2_, which are both bioavailable and energetically favorable electron donors, the microbial populations present in the intervals responded rather quickly, triggering a strong increase in the nitrate reduction kinetics. In Interval 1, introduction of dissolved H_2_ resulted in a longer lag phase compared to the addition of acetate in Interval 2 (5 and 2 days respectively) and an initially slower nitrate reduction before the reaction rate picked up. This suggests that the microbial community present in Interval 1 required more time to adapt to the use of H_2_ and/or autotrophy compared to the use of acetate as electron donor. As Interval 2 had already been injected with acetate previously during preliminary tests, this community was already adapted to a heterotrophic metabolism, which explains its faster response.

The maximal reaction rates were however similar for both electron donors and were up to 60 times higher than the rate observed when the in situ available electron donors were used (Table 3). Furthermore, these reaction rates are also similar to the rates observed during previous injection tests (Table 2 **)** with nitrate and acetate in Interval 2 (i.e. 0.8–1 mM ${\text{NO}}_{3}^{ - }$ per day). The observed stimulation of the nitrate reduction by acetate and H_2_ is in agreement with previous lab tests studying nitrate reactivity in sediments (Devlin et al. 2000; Libert et al. 2011).

In both tests with added electron donors, DNRN and denitrification were the dominant metabolic pathways, which was expected as they are thermodynamically favored over DNRA. In Interval 2 (pulse of acetate), this fast nitrate reduction resulted in an increase in the redox potential of the interval solution, which is in agreement with previously reported lab experiments (Percheron et al. 1998), where an increase in redox potential was observed during sulfide-dependent DNRN and denitrification.

Nitrite accumulated in both tests through DNRN, due to limitations in the amount of electron donor compared to that of the electron acceptor, rendering nitrate reduction more favorable compared to nitrite reduction. Indeed, the C/N ratio after pulse injection of Interval 2 with acetate was 0.4, which is lower than the ratio required to prevent nitrite accumulation and stimulate denitrification (i.e. ~1.2) as was observed during lab experiments (Almeida et al. 1995; Oh and Silverstein 1999). Furthermore, nitrate reductase is reduced preferentially by electrons derived from acetate oxidation compared to nitrite reductase, which also favors the DNRN reaction over the subsequent denitrification pathway (Almeida et al. 1995; Oh and Silverstein 1999), especially when acetate is limited. Nevertheless, the accumulation of nitrite in the interval (up to ~ 10 mM) did not result in inhibition of the microbial reactivity, as was observed for higher concentrations of nitrite (>20 mM) during lab tests (Almeida et al. 1995; Parmentier et al. 2014).

In Interval 1 (pulse of H_2_), nitrite accumulated as well during the fast hydrogenotrophic nitrate reduction, although to a lesser extent than in Interval 2 (pulse of acetate). This accumulation can again be explained by a limitation of the amount of electron donor (i.e. H_2_) compared to the available nitrate, due to the solubility (and thus bioavailability) limit of H_2_. Indeed, previously performed studies (Chang et al. 1999; Haugen et al. 2002; Karanasios et al. 2011) indicated that a decrease of the dissolved H_2_ concentration below a certain threshold (depending on the microbial species and other environmental conditions) would result in a decrease in denitrification rate. Initially, limitation of the hydrogen availability would only inhibit nitrite reductase, resulting in accumulation of nitrite. Lowering the dissolved H_2_ concentration even further would also inhibit the hydrogenotrophic nitrate reduction (Chang et al. 1999).

### Factors influencing the microbial reactions and their rates

The microbial nitrate reduction rates and dominant metabolic pathways observed in the described experiments do not seem to be dependent on the nitrate concentration: similar rates in both intervals and after injection of 1 to 25 mM ${\text{NO}}_{3}^{ - }$ were observed during the preliminary and currently discussed injection tests. Rather the bioavailability and energy yielded by oxidation of the electron donor seem to determine the extent of the nitrate reactivity and the metabolic pathways followed. Note that the reaction rates in the near field of a geological repository for nitrate-containing bituminized radioactive waste are likely also controlled by the release rates of nitrate and acetate from the waste and by the production rate of H_2_ in the repository (by anaerobic corrosion of steel and radiolysis).

### Implications for geological disposal of nitrate-containing waste

The results presented in this paper indicate that microbial nitrate-dependent oxidation of electron donors naturally present in the Opalinus Clay is possible, though occurs only at a slow rate. Acetate leached from the bituminized waste and H_2_ produced by radiolysis of bitumen and water and by anaerobic corrosion of the metallic waste drums could significantly enhance the reaction kinetics. Furthermore, because of the preferential use of these electron donors compared to naturally present electron donors, we do not expect that the reducing capacity of the clay formation would decrease significantly if sufficient amounts of acetate and H_2_ are available. However, a substantial microbially mediated reduction of nitrate with acetate has been shown to increase the redox potential of the pore-water. This could result in less reducing conditions in the pore-water, which might hinder the reduction of redox-sensitive radionuclides (e.g. Se, Tc, U, Np, Pu, etc.), in turn favoring the migration of these radionuclides in the host rock (Oremland et al. 1999; De Cannière et al. 2010).

Furthermore, depending on the electron donor (acetate or H_2_), the denitrification reaction would lead to either a net gas production (of N_2_ in case of heterotrophic reaction with acetate) or gas consumption (of H_2_ in case of hydrogenotrophic reaction). In case the gas generation rate would be higher than its dissipation rate by diffusive transport of the dissolved gas, the former pathway with N_2_ production would result in the formation of a free gas phase, and might therefore give rise to an overpressure with a (local) gas breakthrough, possibly affecting the radionuclide migration. On the other hand, the consumption of H_2_ produced in a repository for bituminized ILW would prevent a gas pressure build-up in the repository and thus a gas-related perturbation of the clay.

## Conclusions

Upon geological disposal of nitrate-containing bituminized ILW in a clay formation, this type of waste will start to take up water, leading to the dissolution of the hygroscopic salts (e.g. NaNO_3_) in the bitumen matrix and subsequently to leaching of NaNO_3_ into the surrounding host rock. Although it remains uncertain whether nitrate-reducing microorganisms can be active under the prevailing harsh conditions for microbes in and around a repository (i.e. high pH, high ionic strength and lack of free space), microbial activity cannot be ruled out. The results obtained by the BN experiment, an in situ experiment in the Opalinus Clay, provide insights in the biogeochemical behavior of nitrate in a clay formation surrounding a repository for nitrate-containing bituminized ILW, in case active microbial species would be present. This paper focused on two injection tests performed in the BN borehole, during which microbial nitrate reactivity was investigated in the presence or in the absence of additional electron donors relevant for such a disposal concept, i.e. acetate (a key bitumen degradation product) and H_2_ (originating from radiolysis and anaerobic steel corrosion).

The results of these tests indicate that nitrate reacts quickly at near neutral pH if external sources of bioavailable and energetically favorable electron donors, such as acetate or H_2_, are present and if enough space and water are available in the system. When only naturally present electron donors are available, nitrate mainly diffuses into the surrounding clay, although also some microbial nitrate reduction (mainly to nitrite) occurs at a slow rate. Both in the presence of H_2_ and acetate, nitrite and nitrogenous gases were predominantly produced, although some ammonium production could also be observed when H_2_ was added. The microbial nitrate reduction rates and dominant metabolic pathways observed in the described experiments are likely depending on the bioavailability and energy-producing capacity of the electron donor present in the system.

The observed reduction of nitrate seems to have an impact on the redox conditions in the pore-water (i.e. an increase of the redox potential), which might affect the migration of radionuclides in the host rock. Furthermore, depending on the electron donor type, denitrification could lead to either a net gas consumption (i.e. oxidation of H_2_) or production (i.e. formation of N_2_) in the repository. The latter might cause an additional stress on the clay, which could lead to gas-related perturbations of the host rock. The observed processes of microbially mediated nitrate reduction should be taken into account during future reactive transport modeling of the migration of (redox-sensitive) radionuclides released from bituminized ILW in the host rock.

Further research is necessary to study the impact of the observed microbial nitrate reduction on the redox state of redox-sensitive radionuclides. In addition, the nitrate reduction rates and production of N species should be investigated further under more realistic repository conditions (e.g. high pH imposed by the use of cementitious materials), to determine the effect of such conditions on the microbial activity and the nitrate reactivity.
